# Mechanical Properties and σ-Phase Precipitation in FeCoCrNiMo_x_ (x = 0, 0.4, 0.5, 0.8, 1.3) High-Entropy Alloys: Insights from First-Principles Study

**DOI:** 10.3390/ma18061267

**Published:** 2025-03-13

**Authors:** Huimin Li, Junjun Jin, Zhiyi Zhang, Jinpeng Yu, Hairong Sun, Songling Sun, Weijie Tang, Guoqing Gou

**Affiliations:** 1 Key Laboratory of Advanced Technologies of Materials, Ministry of Education, School of Materials Science and Engineering, Southwest Jiaotong University, Chengdu 610031, China; swjt_lhm@163.com (H.L.); jinjunjun@my.swjtu.edu.cn (J.J.); 2 CRRC Qingdao Sifang Co., Ltd., Qingdao 266111, China; zhangzhiyi@cqsf.com; 3 School of Computer Engineering, Chengdu Technological University, Chengdu 610031, China; jxsj01330@163.com; 4 Chengdu Metro Operation Co., Ltd., Chengdu 610058, China; shr-19@163.com; 5 Sichuan Special Equipment Inspection Institute, Chengdu 610031, China; cdtj_sunsl@chengdu.gov.cn; 6 Industrial Center, Shenzhen Polytechnic University, Shenzhen 518055, China; tangweijie@szpt.edu.cn

**Keywords:** high-entropy alloys, first-principles calculations, special quasi-random structure, microstructure, mechanical properties

## Abstract

High-entropy alloys (HEAs) have garnered significant global interest due to their outstanding properties. This study investigates the structural stability and mechanical properties of FeCoCrNiMo_x_ (x = 0, 0.4, 0.5, 0.8, 1.3) HEAs using a first-principles approach coupled with the special quasi-random structure (SQS) method. Of the alloys examined, all except FeCoCrNiMo_1.3_ were found to be thermodynamically and dynamically stable. Elasticity calculations revealed that molybdenum improves the ductility and anisotropy of the alloys, though with a slight decrease in strength and stiffness, as confirmed by electronic structure analysis. Defect-free FeCoCrNiMo_0.5_ HEAs coatings were then prepared using laser cladding and characterized for their microstructure and hardness. The coating exhibited a transition from columnar crystals at the bottom to equiaxed crystals at the surface, forming a honeycomb-like structure. Inside the crystal cells, high-density dislocations and σ-phase were observed. Elasticity calculations of the σ-phase confirmed its high hardness, low ductility, and classification as a brittle, hard phase.

## 1. Introduction

High-entropy alloys (HEAs), a novel material class born under a new design paradigm, have fundamentally redefined traditional alloy design principles [1]. In the conventional design framework, the introduction of alloying elements often leads to the formation of multiphase, complex intermetallic compounds. However, multicomponent alloys, characterized by high conformational entropy, tend to reduce Gibbs free energy, thereby favoring the formation of simple solid solutions over complex structures [1,2,3]. This unique behavior has attracted significant attention from researchers worldwide, driven by the exceptional properties of HEAs, such as wear resistance, corrosion resistance, and high strength [4,5,6,7,8].

Among FeCoCrNi-based high-entropy alloys (HEAs), various compositions have shown promising properties for structural applications. For instance, FeCoCrNiMn alloy demonstrates excellent wear resistance [9], while FeCoCrNiCuAl exhibits strong oxidation resistance [4]. The FeCoCrNiMnAl alloy offers high-temperature wear resistance [10], and FeCoCrNiCu alloy provides good dislocation storage capacity as well as high ductility and toughness [11]. Additionally, FeCoCrNiAl_0.5_Ti_0.5_ alloy is noted for its strong corrosion resistance [12].

Molybdenum (Mo), as an alloying element, has been extensively studied for its ability to enhance the mechanical and corrosion-resistant properties of conventional alloys. Dai et al. [13] reported that in FeCoCrNiMo_x_ alloys, Mo content as low as 0.3% led to the formation of precipitates, which increased at higher Mo content, enhancing the protective nature of the passive film and improving corrosion resistance. Furthermore, Zhao [14] demonstrated that increasing Mo content in FeCoCrNiMnMo_x_ alloys led to a transition from a face-centered cubic (FCC) phase to a mixed FCC and body-centered cubic (BCC) phase, significantly improving hardness and reducing the coefficient of friction, thereby enhancing damage tolerance. The effect of Mo content on HEA properties was further exemplified by Gu et al. [15], who found that varying Mo content in Ni_1.5_CrFeTi_2_B_0.5_Mo_x_ HEAs altered the coating structure from a single BCC phase to a dual-phase BCC + FCC structure, improving corrosion resistance in simulated saturated brine mud solutions. Liang [16] designed (FeCoNi)_(88−x)_(AlTi)_12_Mo_x_ (x = 0.5, 1.0, 1.5) HEAs with simple FCC solid solutions, identifying that the Mo1.0 alloy exhibited the best mechanical properties, with yield strength, tensile strength, and elongation reaching 980 MPa, 1260 MPa, and 34.5%, respectively. Additionally, based on valuable research on HEAs, Mo can combine with other elements in the coating to form hard and brittle intermetallic compounds, thereby enhancing the material’s strength and hardness. For instance, Mo and Cr can form the σ phase, which has a body-centered tetragonal structure. These σ phases are dispersed throughout the material, resulting in a precipitation strengthening effect [17].

However, the role of molybdenum in FeCoCrNi-based high-entropy alloys (HEAs) remains underexplored, particularly in optimizing molybdenum content to balance mechanical strength, ductility, and thermal stability. While earlier studies have focused on molybdenum-induced phase transitions and hardness improvements, there is still a lack of detailed understanding regarding the relationship between molybdenum content, microstructure, mechanical properties, and electronic structure in HEAs. Most existing research, both experimental and computational, has concentrated on molybdenum contents up to 0.3 [18,19], with limited exploration of higher molybdenum fractions (e.g., 0.4, 0.5, etc.). The effects of these higher concentrations, particularly in combination with other elements, remain largely unexplored. Therefore, this paper presents a comprehensive study of FeCoCrNiMo_x_ (x = 0.0, 0.4, 0.5, 0.8, 1.3) alloys using first-principles methods based on density functional theory (DFT), investigating the effect of varying molybdenum content on the structural stability, mechanical properties, and electronic structure of FeCoCrNiMo_x_ HEAs. Additionally, based on the results of first-principles calculations, a FeCoCrNiMo_0.5_ coating was fabricated using laser cladding, and its microstructure and mechanical properties were characterized. This study contributes to the growing body of knowledge on HEAs and highlights the potential of molybdenum-doped FeCoCrNi-based alloys for engineering applications that demand high strength and thermal stability.

## 2. Methods

### 2.1. Computational Details

**Figure 1:**
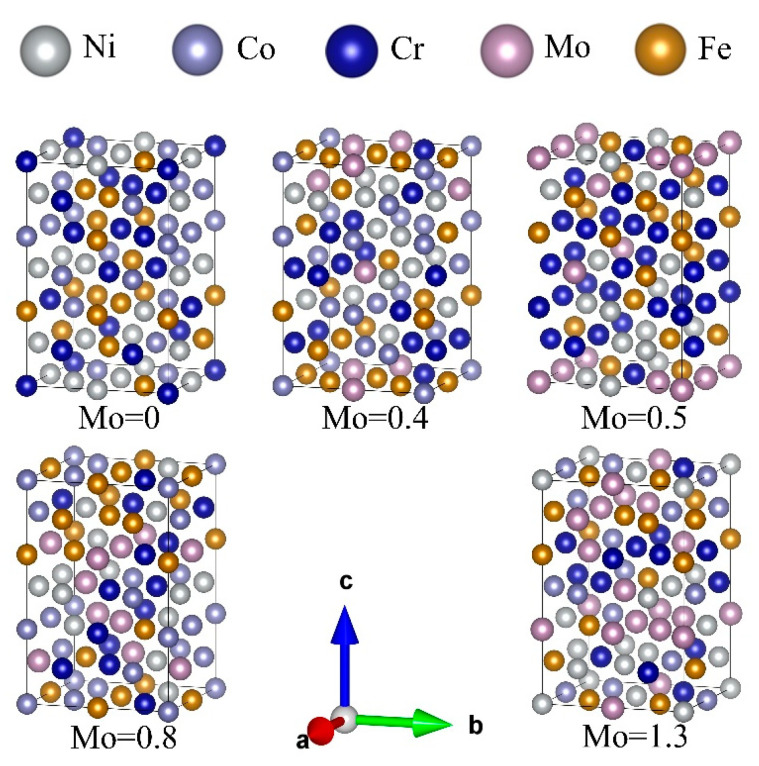
The 2 × 2 × 3 SQS supercell for different Mo elemental contents.

To compute the elastic constants of FeCoCrNiMo_x_ HEAs using the first-principles approach, we employed the Monte Carlo SQS method [20] to generate a symmetrically ideal FCC lattice with site occupancies for the FeCoCrNiMo_x_ (x = 0, 0.4, 0.5, 0.8, 1.3) HEAs model. The FCC structure is determined by the atomic radius difference (δ) and valence electron concentration (VEC) in Table 1. When the atomic radius difference is less than 6.6%, and the valence electron concentration is greater than 8, the structure of high-entropy alloys mainly consists of the FCC structure. To accommodate our computational resources, we adopted a compromise solution by constructing a 2 × 2 × 3 supercell with FCC structure, each containing 48 atoms, and the SQS model for each component is illustrated in Figure 1.

All first-principles computational methods based on density functional theory utilize the generalized gradient approximation (GGA) of the Perdew–Burke–Ernzerhof (PBE) generalization as the exchange-correlation functional for self-consistent calculations [22]. To ensure computation accuracy, we conducted a rigorous convergence test by computing Brillouin zone integrals. The convergence criteria were set as follows: stress less than 0.01 GPa, force tolerance less than 0.005 eV/Å, maximum displacement less than 0.0003 Å, and total energy change less than 3 × 10^−6^ eV/atom. After the convergence test, a final plane wave energy cutoff of 500 eV and a 3 × 3 × 2 k-point mesh were selected to calculate the elastic constants of the HEA. The energy variation with increasing cutoff energy and k-points was less than 1 meV/atom. Before calculating the elastic constants, all structures were optimized and energetically computed using the CASTEP software (Version 2019). The equilibrium lattice constant of the FeCoCrNi HEA after structural optimization was 3.507 Å, which aligns well with previous calculations and experimental data (a = 3.568 Å [14], a = 3.540 Å [23]), with an error of less than 2%. This suggests the reliability of our calculations. The dynamic stability of FeCoCrNiMo_x_ HEAs was examined by the phonon dispersion curves. The elastic stiffness constants were then calculated using the stress–strain method [24], and the associated mechanical properties were determined.

### 2.2. Experimental Method

Based on the calculations, the composition at x = 0.5 optimizes the balance between strength and ductility, and therefore, the FeCoCrNiMo0.5 coatings were prepared by laser deposition. This study used quenched and tempered EA4T axle steel as the base material. The coating material, FeCoCrNiMo_0.5_ HEAs powder, was produced through vacuum atomization. Before laser cladding, the powder was dried in an oven at 120 °C for 2 h to enhance its flowability. The FeCoCrNiMo_0.5_ HEAs coating was applied using the laser cladding method with the following parameters: laser power of 1800 W, scanning speed of 300 mm/min, spot diameter of 5 mm, powder feeding speed of 2 r/min, and a lap rate of 50%. Specimens were cut perpendicular to the coating using a linear cutting method. The specimens for microstructural analysis were ground using 400 to 2000 grit silicon carbide paper, polished with 1 μm diamond powder, and electrolytically etched with a 5% oxalic acid solution. The coating microstructure was examined using electron microscopy, X-ray diffraction, scanning electron microscopy, and electron channel contrast imaging (ECCI). The compositional distribution was analyzed using energy dispersive spectroscopy (EDS). A JMHVS-1000AT digital microhardness tester (Aolong Company, Shanghai, China) was used to measure the microhardness of the coating and substrate. The test parameters were as follows: a loading force of 1.96 N, a holding time of 15 s, and test areas including the coating, bonding zone, heat-affected zone, and substrate. To visualize the hardness variations across different areas of the cladding specimen, tests were performed at equal intervals from the coating to the substrate, with a 0.2 mm spacing between each test point. In this study, residual stresses across different areas of the coating cross-section were measured using a μ-X360s X-ray residual stress analyzer (Pulstec Company, Shizuoka, Japan). The cosα method was employed for stress calculation, utilizing diffraction data collected from the diffraction ring (normal strain in the direction of the Debye ring circumference angle) using a two-dimensional detector. The analysis employed a Cr target; the diameter of the collimating tube was 1 mm, power setting at 30 kV·1 mA, with a zero stress iron powder calibration. Following a single incident at a single angle, a complete Debye ring was obtained, where the difference between the Debye ring without stress and the deformed Debye ring under stress facilitated calculation of the change of interplanar spacing under stress and the corresponding stress. This approach provided information on full residual stress. The KEYSIGHT G200 Nano Indenter (Santa Rosa, CA, USA) was used to conduct nanoindentation tests on the coatings, using a diamond Berkovich indenter and controlling the indentation-depth mode. The specific test parameters were set as follows: maximum indentation depth of 500 nm, loading strain rate of 1 mN/s, holding time of 10 s to reach the maximum indentation depth, and then unloading.

## 3. Results and Discussion

### 3.1. First-Principles Calculation

#### 3.1.1. Thermodynamic and Dynamic Stability Analysis

**Figure 2:**
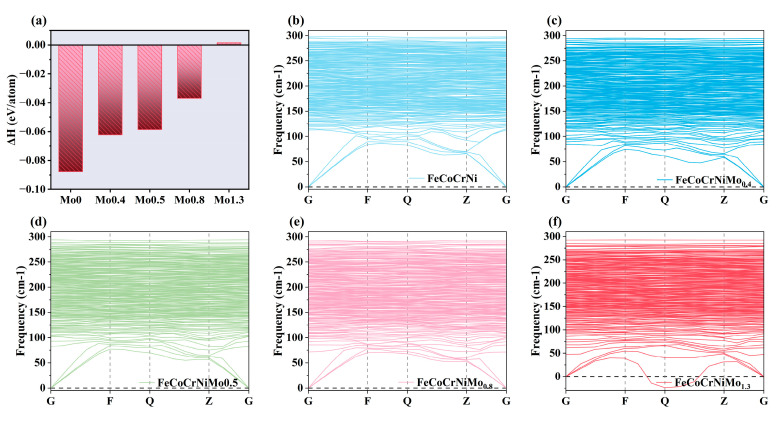
(**a**) Formation enthalpy; (**b**–**f**) phonon dispersion curves: (**b**) FeCoCrNi; (**c**) FeCoCrNiMo_0.4_; (**d**) FeCoCrNiMo_0.5_; (**e**) FeCoCrNiMo_0.8_; (**f**) FeCoCrNiMo_1.3_.

To investigate the structural characteristics of FeCoCrNiMo_x_ HEAs, we first calculated the formation enthalpies of four alloys to determine their thermodynamic stability [25]. The formation enthalpy serves as a key indicator of the thermodynamic stability of HEAs, representing the enthalpy change when 1 mol of the substance is formed from its constituent elements in their standard states. This process involves the rearrangement of atoms and the formation of new chemical bonds, leading to changes in bond energy, van der Waals forces, and electrostatic energy, which collectively result in a change in the system’s total enthalpy. The formula for calculating the formation enthalpy ($\Delta H_{f}$) is as follows:

(1) $$\Delta H_{f}=\frac{1}{x+y+z+m+n}\left[E_{FeCoCrNiMo_{x}}-\left(xE_{Fe}+yE_{Co}+zE_{Cr}+mE_{Ni}+nE_{Mo}\right)\right]$$

where E_FeCoCrNiMox_ is the total energy of FeCoCrNiMo_x_ models. E(Fe), E(Co), E(Cr), E(Ni), and E(Mo) represent the total energies of Fe, Co, Cr, Ni, and Mo, respectively, while x + y + z + m + n corresponds to the total number of atoms in the model. The energies of FeCoCrNiMo_x_ were calculated using first-principles density-functional theory (DFT). These calculations were performed with the CASTEP software for each alloy model. Structural optimization was carried out to minimize atomic forces, ensuring accurate ground-state energy representations for each component. For the five pure metals (Fe, Co, Cr, Ni, and Mo), we considered their respective crystal structures—FCC for Ni, Cr, and Co and BCC for Fe and Mo—and modeled them separately. The energy calculations for these pure metals followed the same approach used for the FeCoCrNiMo_x_ alloy. A negative formation enthalpy indicates thermodynamic stability, and the more negative the formation enthalpy, the easier the formation process and the more stable the phase. Figure 2a shows the formation enthalpies of the five alloys. Except for the alloy with a Mo content of 1.3, the formation enthalpies of the other four alloys are negative. The alloy without molybdenum exhibited the lowest enthalpy of formation, approximately −0.08 eV/atom, which corresponds well with the calculation by Hayun et al. (−0.036 ± 0.01 eV/atom) [26]. As the molybdenum content increases, the thermodynamic stability gradually decreases, which is consistent with the findings of Cichocki et al. [19].

To further determine the stability of the five alloys, we also calculated the phonon dispersion curves to assess their dynamic stability. Dynamic stability is determined by the characteristics of the phonon frequencies; if all frequencies in the phonon dispersion curve are positive, the phase is considered dynamically stable [27,28]. Conversely, the presence of imaginary phonon frequencies indicates dynamic instability. The phonon dispersion curves for the four alloys are shown in Figure 2b–f. The results indicate that FeCoCrNiMo_1.3_ exhibits imaginary phonon frequencies, suggesting dynamic instability, whereas the phonon frequencies for the other four alloys are all positive, indicating the presence of dynamic stability. In summary, combining these results with the formation enthalpy analysis, we conclude that FeCoCrNiMo_1.3_ cannot stably exist. This can be attributed to the higher concentration of Mo in the alloy, which could disrupt the ideal atomic arrangement required for stability in high-entropy alloys (HEAs). The large atomic size difference between Mo and the other elements may also contribute to lattice distortions, leading to higher enthalpy. Therefore, it is not further discussed in this paper.

#### 3.1.2. Effect of Mo Content on Mechanical Properties

For the structurally optimized model, the elastic constants C_ij_ of the alloys, as calculated by the stress–strain method of generalized Hooke’s law [29,30], can be used to assess their mechanical properties. According to Born–Huang theory, for face-centered cubic crystals, the mechanical stability is determined by the following conditions [31]:

C_11_ > 0, C_44_ > 0, C_11_ − C_12_ > 0, C_11_ + 2C_12_ > 0 (2)

**Figure 3:**
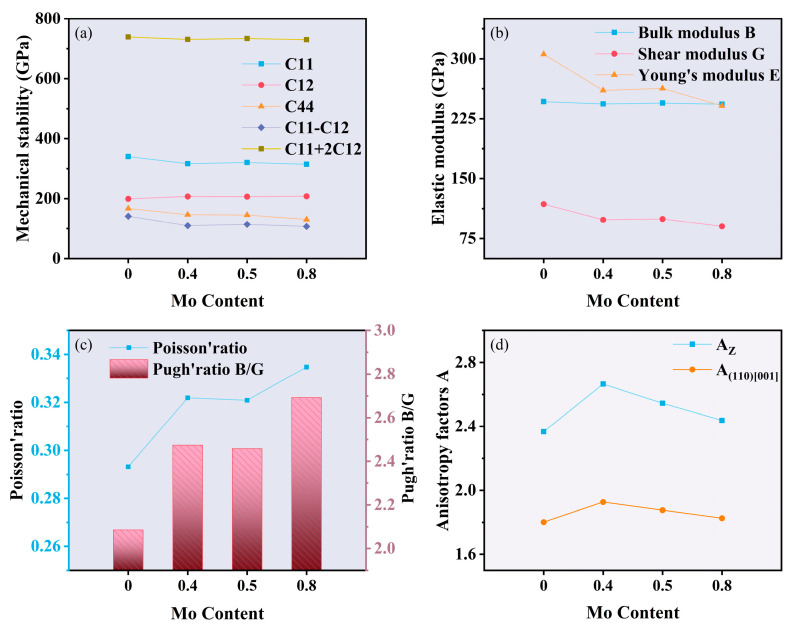
Mechanical properties at different Mo contents: (**a**) variation of elastic constants with Mo content; (**b**,**c**) variation of mechanical properties with Mo content; (**d**) variation of anisotropy with Mo content.

In Figure 3a, it is observed that all compositions of HEAs satisfy the mechanical stability conditions. Notably, C_11_ and C_44_ demonstrated a significant decrease with increasing Mo content, while C_12_ slightly increased. C_11_ represents the linear compressive response in the a-axis, and C_44_, which is related to its shear behavior, is the key parameter affecting the hardness. Thus, the addition of Mo elemental content in the alloy decreased the compressive resistance and hardness of the alloy.

Based on the results of the elastic constants, the elastic properties of HEAs, including bulk modulus, shear modulus, Young’s modulus, and Poisson’s ratio, can be determined using the Voigt–Reuss–Hill approximation [32]. Since the Voigt and Reuss approximations denote the maximum and minimum limits of the polycrystalline elastic modulus, respectively, the bulk modulus B and shear modulus G can be expressed as the Voigt and Reuss averages between them. For the cubic phase, B and G can be given by the following equations [33]:

(3) $$B=\frac{1}{3}(C_{11}+2C_{12})$$

(4) $$G=\frac{1}{2}(G_{V}+G_{R})$$

where Voigt shear modulus $G_{V}=(C_{11}-C_{12}+3C_{44})/5$, and Reuss shear modulus $G_{R}=5(C_{11}-C_{12})C_{44}/\left[4C_{44}+3(C_{11}-C_{12})\right]$.

In addition, Young’s modulus and Poisson’s ratio can be obtained from B and G [34]:

(5) $$E=\frac{9BG}{3B+G}$$

(6) $$V=\frac{\left(3B-2G\right)}{2(38+G)}$$

The calculated elastic properties of the four alloys are shown in Figure 3b,c. The bulk modulus B of the alloy is usually proportional to the cohesive energy of the material, which represents the resistance of the material to bond fracture. The shear modulus G and Young’s modulus E represent the resistance of the material to shear deformation and the stiffness of the material [35], respectively, and can be used to assess the mechanical hardness of the alloy in the annealed state. Meanwhile, the Poisson’s ratio can be used to assess the degree of covalent bonding and to predict its ductility or brittleness. When the Poisson’s ratio is greater than 0.26, the material is more ductile [36]. In addition, the ratio of bulk modulus to shear modulus (B/G) is also closely related to the ductility of the alloy. When the ratio is greater than 1.75, it means that the material is more ductile [37]. From the calculation results, it can be seen that both the shear modulus and Young’s modulus decreased with the increase in Mo content, while Poisson’s ratio and B/G showed the opposite trend. Poisson’s ratio was greater than 0.26, and B/G was greater than 1.75, and the bulk modulus was basically unchanged. These results indicate that the addition of Mo element improved the ductility of the alloy but at the same time reduced its shear resistance as well as its stiffness.

Typically, applications of crystalline materials are significantly affected by mechanical anisotropy. The elastic anisotropy of a material is assessed by means of an anisotropy factor A, which is also used to study the mechanical properties of anisotropic materials, where A = 1 indicates isotropy and otherwise anisotropy. The magnitude of A determines the degree of anisotropy, and the greater the deviation of A from 1, the more anisotropic the material is. Based on the elasticity constant C_ij_ for cubic crystals, we calculated the Zener anisotropy ratio A_Z_ and the anisotropy factor A _(110) [001]_ by means of Equations (6) and (7), where the Zener ratio A_Z_ denotes the ratio of the shear moduli between the (100) and [110] crystal planes, which can be used to characterize the degree of anisotropy of the material:

(7) $$A_{Z}=\frac{2C_{44}}{C_{11}-C_{12}}$$

(8) $$A_{(110)\left[001\right]}=\frac{C_{44}(C^{’}+2C_{12}+C_{11})}{C_{11}C^{’}-C_{12}^{2}}$$

where $C^{’}=C_{44}+(C_{11}+C_{12})/2$. A _(110) [001]_ denotes the anisotropy factor in the (110) [001] direction. By using the above equations, we calculated the Zener ratios A_Z_ and anisotropy factors A _(110) [001]_ for the four alloys, and the results are shown in Figure 3d, and the detailed values are presented in Table 2. The Zener ratio AZ and anisotropy factor A _(110) [001]_ of all the alloys were not equal to 1, which indicates that the HEAs of all four compositions are anisotropic materials. The Zener ratio AZ and anisotropy factor A _(110) [001]_ showed the same increasing trend with increasing Mo content.

**Figure 4:**
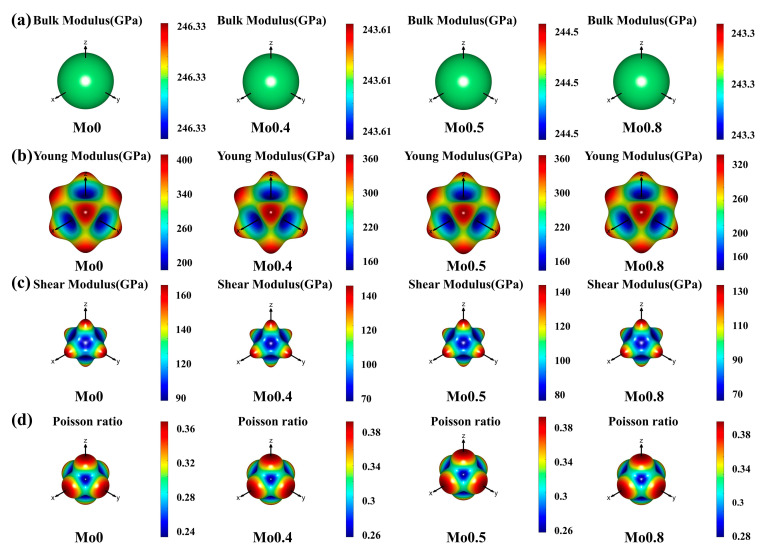
Three-dimensional surface plot: (**a**) Bulk modulus; (**b**) Young’s modulus; (**c**) shear modulus; (**d**) Poisson’s ratio.

In order to examine the anisotropic properties of the four alloys, the three-dimensional surfaces representing the Bulk modulus, Young’s modulus, shear modulus, and Poisson’s ratio were further analyzed. An alloy demonstrates isotropy when its three-dimensional surface manifests as a sphere; otherwise, it displays anisotropic behavior. Greater deviation of the 3D surface from a sphere signifies higher levels of anisotropy. Figure 4 depicts the three-dimensional surfaces of the bulk modulus B, Young’s modulus E, shear modulus G, and Poisson’s ratio V, respectively. Inspection of Figure 4a reveals that the 3D surfaces representing the bulk modulus of the four alloys conform to standard spherical shapes, indicating isotropic behavior. However, in Figure 4b–d, the shapes of the surfaces representing Young’s modulus, shear modulus, and Poisson’s ratio for the four alloys deviate significantly from that of a standard sphere, suggesting pronounced anisotropy. The color of a three-dimensional surface corresponds to the magnitude of its modulus, while its curvature indicates the extent of anisotropy. Young’s modulus surfaces for all alloys exhibit eight corners, whereas the shear modulus and Poisson’s ratio surfaces possess six corners. Furthermore, the maximum values of Young’s modulus align with the <111> direction, whereas for shear modulus and Poisson’s ratio, they align with the <100> direction. This alignment suggests that the alloys are more resistant to longitudinal deformation along the <111> direction and transverse deformation along the <100> direction.

#### 3.1.3. Effect of Mo Content on Electronic Structure

**Figure 5:**
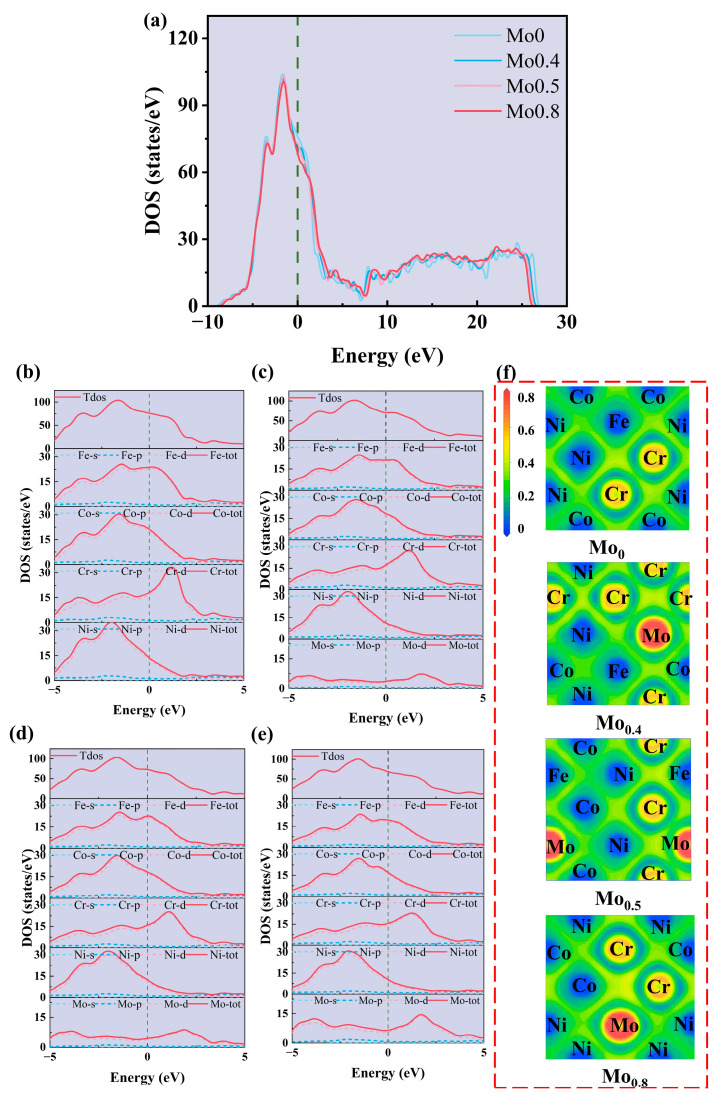
(**a**) Total density of states of FeCoCrNiMo_x_; (**b**–**d**) partial density of states of FeCoCrNiMo_x_: (**b**) Mo_0_, (**c**) Mo_0.4_, (**d**) Mo_0.5_ and (**e**) Mo_0.8_. (**f**) Contour plots of ELF on the (1 0 0) plane of FeCoCrNiMo_x_.

The electronic structure significantly influences the stability and mechanical properties of materials. To elucidate the relationship between the mechanical properties of FeCoCrNiMo_x_ high-entropy alloys (HEAs) and Mo elements from an electronic perspective, we analyzed changes in the density of states (DOS) resulting from the introduction of Mo elements, as depicted in Figure 5a–d. The position of the Fermi energy level is denoted by the green dashed line. Figure 5a illustrates the total density of states (TDOS), indicating the metallic nature of FeCoCrNiMo_x_ with a non-zero density at the Fermi energy level. As Mo content increases, the TDOS at lower energy levels diminishes, with peak positions shifting towards higher energy levels, signifying a gradual reduction in the alloy’s structural stability [38]. Simultaneously, the depth of the pseudo-energy gap near the Fermi energy levels in the TDOS decreases with increasing Mo content, implying a weakening of atomic covalency and consequent reduction in mechanical properties [39,40].

Furthermore, as depicted in Figure 5b–d, analysis of the partial density of states (PDOS) for each metallic element reveals a notable hybridization of electronic states within the −5–5 eV range. Particularly, the d orbitals of each element predominantly contribute to these electronic states, with the electron occupation of these d-orbitals significantly impacting the materials’ mechanical properties [41]. The height of the DOS peak of Mo notably escalates with increasing Mo content, particularly for the first peak above the Fermi energy level, albeit consistently lower than peaks of other elements. The diminished hybridization strength between Mo’s d orbitals and those of other elements results in the formation of covalent bonds with reduced strength compared to bonds involving other elements. The weaker covalent bonding could make the material more prone to brittle fracture, especially under tensile stress or high strain rates. This is because materials with weaker covalent bonds generally exhibit lower resistance to crack propagation. Consequently, the system’s mechanical properties exhibit a decline with increasing Mo content.

To explore the correlation between electronic structure and mechanical properties in alloys, electronic localization functions (ELF) were computed for alloys with varying compositions. ELF offer both quantitative and visual insights into the chemical environment of a compound. For instance, an ELF value of 0.5 signifies metallic bonding properties, while a value of 1 indicates full covalent bonding. Figure 5e illustrates the ELF plot of FeCoCrNiMo_x_. In the FeCoCrNi HEA, the electron cloud is uniformly distributed around Fe, Co, and Ni, exhibiting similar electronegativities and an ELF value of approximately 0.35, indicating typical metallic bonding. Conversely, a charge depletion region (depicted in light blue) surrounds Mo and Cr atoms due to their lower valence electron counts, leading to electron expulsion from their surroundings. This phenomenon weakens the bonds between Cr/Mo and their nearest neighboring atoms. Consequently, as Mo content increases, interlayer bonds become more susceptible to breakage, rendering Mo-containing alloys more prone to slip, thereby diminishing their mechanical properties.

### 3.2. Microstructure and Microhardness

**Figure 6:**
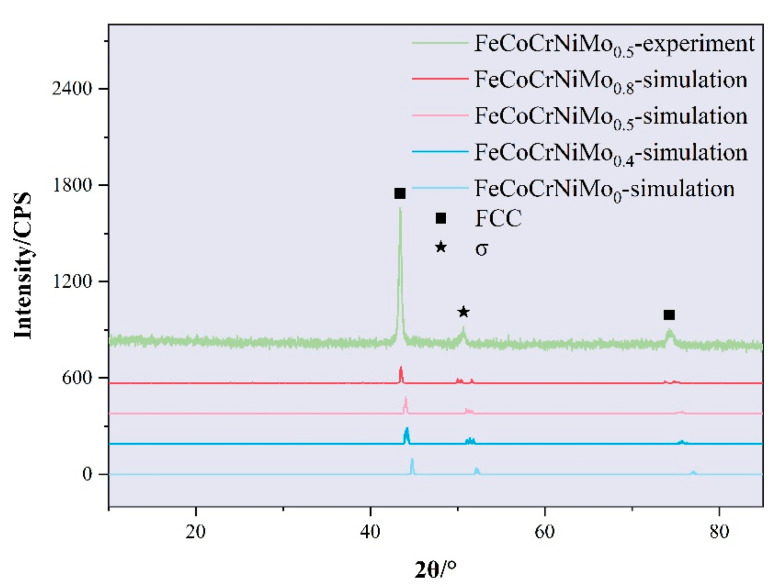
X-ray diffraction results of FeCoCrNiMo_x_ HEA.

Figure 6 presents the X-ray diffraction results of FeCoCrNiMo_x_ HEA. First-principles-calculated XRD patterns of the four high-entropy alloys revealed a dual-phase structure dominated by face-centered cubic (FCC) phases with minor σ-phase contributions. By comparing the positions of the diffraction peaks, it can be observed that the diffraction peaks as a whole show a slight leftward shift with the addition of Mo element. This suggests that the introduction of Mo element leads to significant lattice distortion, causing the lattice constant to increase with its content. Similar to the results of first-principles calculations, the phase structure of the FeCoCrNiMo_0.5_ coating mainly consists of the FCC phase and the σ phase, which is the same as that observed by Zhao et al. [42]. The addition of Mo promotes the formation of sigma phase but did not change the crystal structure (FCC). The σ-phase formation is primarily due to molybdenum’s relatively low enthalpy of mixing with other elements, making it prone to combining with them. The addition of molybdenum enhances the tendency for intermetallic compound formation, and the segregation of Mo and Cr promotes the formation of the σ phase [43]. Molybdenum’s low enthalpy of formation with metals like Fe, Ni, and Co favors the energetically favorable mixing of these metals into a solid solution. Despite molybdenum’s larger atomic radius and higher melting point, these thermodynamic properties—especially the favorable interactions with other alloying elements—overcome size differences and promote its solubility in the HEA system. Furthermore, the high configurational entropy resulting from the near-equal mixing of multiple elements stabilizes phases like FCC solid solutions and complex intermetallics. This entropy effect counteracts the tendency of immiscible elements to phase separate.

**Figure 7:**
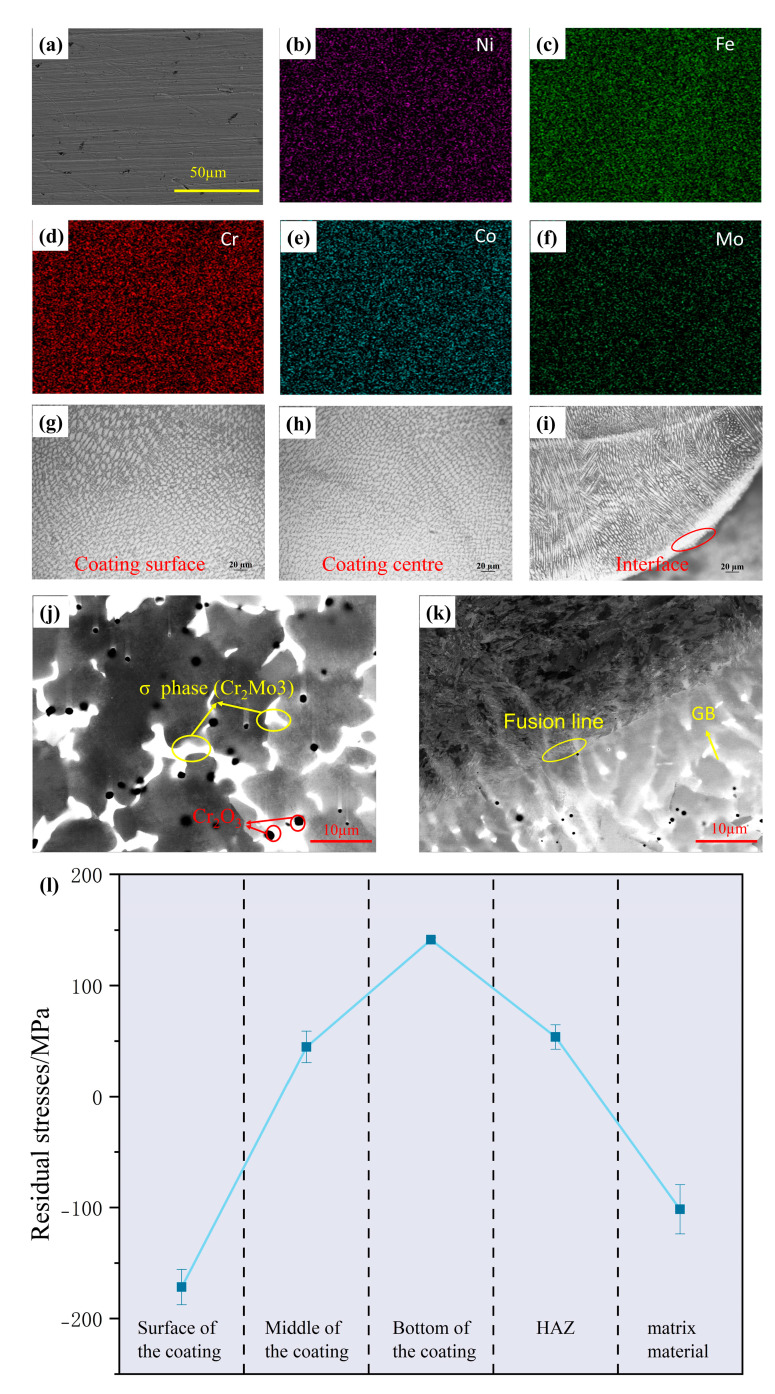
Microstructure of FeCoCrNiMo_0.5_ HEA coating: (**a**–**f**) secondary electron images and EDS mappings of coating; (**g**–**i**) coating’s microstructure at the surface, middle, and interface; (**j**,**k**) electron channeling contrast imaging (ECCI) at the coating surface and interface; (**l**) residual stress distribution in coatings.

Figure 7a–f display secondary electron images and EDS mappings of FeCoCrNiMo_0.5_ HEA coating. The images reveal a defect-free and crack-free surface, with uniform distribution of the five elements—Fe, Co, Cr, Ni, and Mo—across the coating. No signs of elemental segregation are observed. Figure 7g–i illustrate the coating’s microstructure at the surface, middle, and interface. The surface consists primarily of equiaxed crystals, while the middle includes both equiaxed and columnar crystals. The bottom is characterized by columnar crystals. Overall, the coating transitions from columnar to equiaxed crystals, forming a honeycomb-like structure [43]. The formation of columnar grains near the substrate and equiaxed grains at the surface leads to differences in thermal expansion, which can induce internal stresses upon cooling. This phenomenon, commonly observed in coatings produced by rapid cooling techniques such as laser cladding and welding, may adversely affect mechanical properties, particularly fatigue resistance, and could lead to cracking under mechanical loading. To investigate the distribution of these residual stresses, tests were conducted on different regions of the coating, as shown in Figure 7l. The results demonstrate that the surface of the coating experiences residual compressive stress due to rapid cooling, which restricts thermal contraction. The middle region shows minimal residual tensile stress, while the bottom exhibits significant residual tensile stress. This is attributed to the large mismatch in the thermal expansion coefficients and the growth direction of columnar crystals between the coating and the substrate. Additionally, phase expansion of the substrate induces a reverse constraint on the coating, resulting in high residual tensile stress at the bottom, which progressively decreases towards the heat-affected zone. Figure 7j,k present electron channeling contrast imaging (ECCI) at the coating surface and interface. The images reveal closely aligned crystal cells and white precipitates. A high density of dislocations is present within the crystal cells, while the white precipitates, identified as Mo-rich σ phase (Cr_2_Mo_3_), are located at grain boundaries and within the cells [44,45,46]. The combination of these dislocations and the precipitated σ phase are the key for the increase in the hardness of the coating [17]. Additionally, black particles, primarily Cr_2_O_3_ oxides, are found at grain boundaries and within the grains. These oxides form during the laser cladding process.

**Figure 8:**
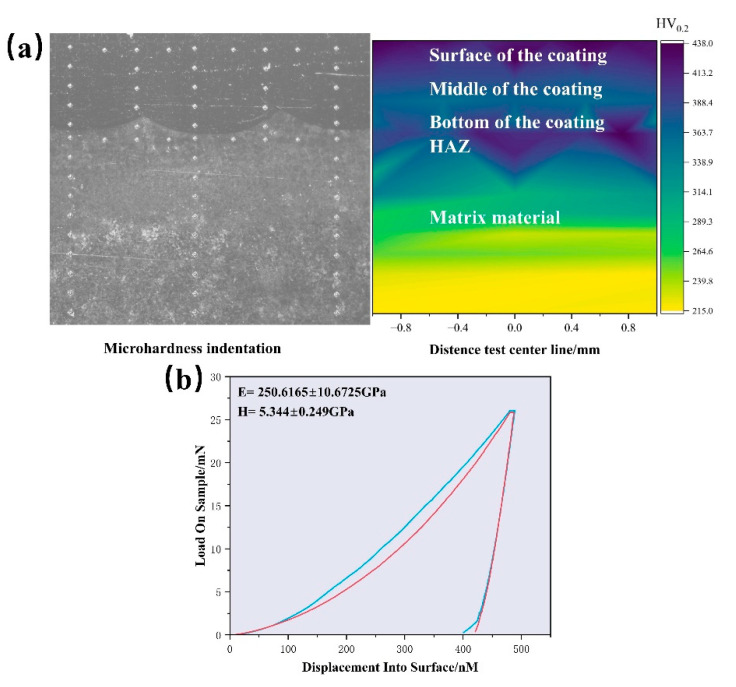
Mechanical properties of FeCoCrNiMo0.5 HEA coating: (**a**) Vickers hardness indentation and hardness values of coating cross-sections; (**b**) nanoindentation results for coating.

The indentation and hardness values across the cross-section of the FeCoCrNiMo_0.5_ HEA are shown in Figure 8a. The hardness increases progressively from the substrate to the coating, forming a clear gradient. The microhardness distribution diagrams of the substrate, cladding layer, and heat-affected zone show that the EA4T steel substrate retains its original material properties, with an average hardness of 218.9 HV_0.2_. The heat-affected zone is relatively narrow, and its hardness varies significantly. Near the interface, the hardness is close to that of the coating, but as the distance from the interface increases, the hardness gradually decreases, approaching the substrate’s value. The microhardness in the heat-affected zone, compared to the substrate, is largely influenced by thermal cycling. The formation of residual austenite also contributes to the hardness increase. The maximum hardness of the coating is 437.91 HV_0.2_, with the surface being significantly harder than the bottom. This hardness variation is primarily due to differences in microstructure: the surface consists of fine equiaxed crystals, while the bottom is mainly made up of coarse columnar crystals. Figure 8b shows typical indentation load–depth curves of the FeCoCrNiMo_0.5_ HEA. The average Young’s modulus and hardness of the alloy are 250.6 and 5.3 GPa, respectively. The value of Young’s modulus measured by the nanoindentation has a good correspondence with the previous first-principle calculation (262.8 GPa), with an error of about 4.9%. This also verifies the accuracy of our calculation results from the side.

### 3.3. Elastic Properties of σ Phase

**Figure 9:**
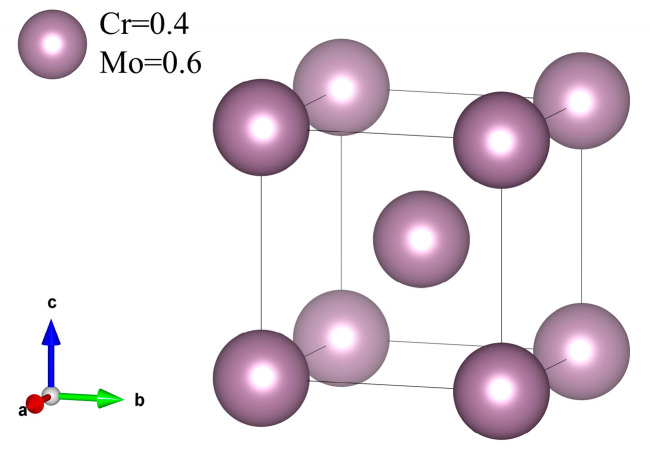
Model structure of the σ phase.

Section 3.2 highlights the significant presence of the σ phase in the coating, which greatly impacts the mechanical properties of FeCoCrNiMo_0.5_ HEA. Therefore, it is essential to investigate the elastic properties of the σ phase to understand its influence on the alloy’s overall behavior. The σ phase is primarily composed of Cr_2_Mo_3_, which has a cubic structure. To analyze it, a crystal structure model of Cr_2_Mo_3_ was created, as illustrated in Figure 9. After optimizing the structure, the elastic properties were calculated using the stress–strain method based on first-principles calculations.

The mechanical stability conditions for the cubic crystal system, as defined by the Born–Huang theory, are given in Equation (2). Table 2 shows that the elastic constants of the σ phase meet the mechanical stability criteria, confirming that the phase is structurally stable. Elastic property calculations indicate that the bulk modulus (B) and shear modulus (G) are low compared to the FCC phase, with a Poisson’s ratio below 0.26 and a B/G ratio less than 1.75. This suggests that the σ phase has weak shear resistance and low ductility, making it an entirely brittle phase. Using Chen et al.’s hardness prediction formula [47], the hardness of the σ phase was calculated to be approximately 22.7 GPa, which corresponds to a Vickers hardness of about 2300 HV. This value is significantly higher than that of conventional alloys. Therefore, the σ phase, formed due to the addition of Mo, is a brittle and hard phase that significantly alters the coating’s properties and enhances its hardness. Moderate amounts of the σ phase can significantly enhance the hardness of the alloy, whereas excessive precipitation of the σ phase leads to increased brittleness [48].

To mitigate the brittleness while maintaining the hardness benefits of the σ phase, strategies such as heat treatment (e.g., annealing) or compositional adjustments can be considered. Heat treatment may modify the distribution and morphology of the σ phase, potentially reducing its negative impact on ductility. For example, the formation of the σ phase can be suppressed by controlling the cooling rate and introducing an annealing step, which enhances the ductility of the alloy without significantly reducing the hardness [49]. Additionally, compositional adjustments, such as reducing Mo content or adding stabilizing elements like Ti, can suppress σ-phase formation or modify its morphology [48].

## 4. Conclusions

This study investigated the mechanical properties and electronic structure of FeCoCrNiMo_x_ HEAs using first-principles calculations based on density functional theory. Additionally, FeCoCrNiMo_0.5_ HEAs coatings were prepared on EA4T steel using the laser cladding method, and their microstructure and microhardness were characterized. The main conclusions of the study are as follows:

(1) The addition of molybdenum increases the ductility and anisotropy of the FeCoCrNiMo_x_ HEAs FCC phase, though it slightly reduces strength and stiffness, as confirmed by electronic structure calculations. As the Mo content increases, lattice distortion in the coating becomes more pronounced, with a corresponding leftward shift in the diffraction peak. The Young’s modulus measured by nanoindentation closely aligns with first-principles calculations, showing a deviation of approximately 4.9%;
(2) The FeCoCrNiMo_0.5_ HEAs coatings exhibit a transition from columnar crystals at the bottom to equiaxed crystals at the surface. The surface hardness reaches 437.91 HV_0.2_, while the bottom shows slightly lower hardness. Residual stress tests show that the coating surface experiences residual compressive stress due to rapid cooling, while the bottom exhibits significant residual tensile stress. This is attributed to the thermal expansion coefficient mismatch and the directional growth of columnar crystals between the coating and substrate. Furthermore, a large number of σ phases were precipitated in the coating due to the deviation of Cr and Mo elements;
(3) The elastic properties of the σ phase were calculated using the stress–strain method, showing low bulk modulus, shear modulus, and Poisson’s ratio. Its hardness is 22.7 GPa, classifying it as a brittle, hard phase. The presence of the σ phase alters the coating’s properties, contributing to its increased hardness.

## Figures and Tables

**Table 1:** The calculation results of δ and VEC in this system.

	δ%	VEC
FeCoCrNiMo_0_	5.54	8.2500
FeCoCrNiMo_0.4_	5.41	8.0625
FeCoCrNiMo_0.5_	5.39	8.0624
FeCoCrNiMo_0.8_	5.27	7.8749
FeCoCrNiMo_1.3_	5.13	7.6875

When δ ≤ 6.6%, it is beneficial to form a single solid solution; when VEC ≥ 8, it tends to form FCC structure phase [21].

**Table 2:** Elastic constants and properties of σ phase.

	Elastic Properties						Elastic Constants		
	B	E	G	H	V	B/G	C11	C12	C44
Σ phase	113.1	239.9	104.7	22.7	0.15	1.1	240.9	49.1	110.9

## Data Availability

The original contributions presented in this study are included in the article. Further inquiries can be directed to the corresponding author.
